# Nonimmunogenetic Viral Capsid Carrier with Cancer Targeting Activity

**DOI:** 10.1002/advs.201800494

**Published:** 2018-06-27

**Authors:** Bo‐Ram Lee, Eunji Jo, Hong Yeol Yoon, Chul Joo Yoon, Hyo‐Jung Lee, Koo Chul Kwon, Tae Woo Kim, Jeewon Lee

**Affiliations:** ^1^ Department of Chemical and Biological Engineering College of Engineering Korea University Anam‐Ro 145 Seoul 136‐713 Republic of Korea; ^2^ Center for Theragnosis Biomedical Research Institute Korea Institute of Science and Technology 39‐1 Hawolgok‐dong, Seongbuk‐gu Seoul 136‐791 Republic of Korea; ^3^ Division of Infection and Immunology Graduate School of Medicine Korea University Anam‐Ro 145 Seoul 136‐713 Republic of Korea

**Keywords:** albumin, albumin‐binding peptides, immunogenicity, protein nanoparticles, tumor targeting, viral capsids

## Abstract

Although protein nanoparticles (PNPs) (e.g., viral capsids) capable of delivering a broad range of drug agents have shown distinctive advantages over synthetic nanomaterials, PNPs have an intrinsic drawback that hampers their clinical application, that is, potential immunogenicity. Here, a novel method for resolving the immunogenicity problem of PNPs, which is based on the genetic presentation of albumin‐binding peptides (ABPs) on the surface of PNP, is reported. ABPs are inserted into the surface of a viral capsid (hepatitis B virus capsid/HBVC) while preserving the native self‐assembly function of HBVC. The ABPs effectively gather human serum albumins around HBVC and significantly reduce both inflammatory response and immunoglobulin titer in live mice compared to ABP‐free HBVC. Furthermore, ABP‐conjugated HBVCs remain within tumors for a longer period than HBVCs conjugated to tumor cell receptor‐bindingpeptides, indicating that the ABPs are also capable of enhancing tumor‐targeting performance. Although applied to HBVC for proof of concept, this novel approach may provide a general platform for resolving immunogenicity and cancer‐targeting problems of PNPs, which enables the development of a variety of PNP‐based drug delivery carriers with high safety and efficacy.

## 1 Introduction

Reportedly, hepatitis B virus capsid (HBVC) has a great potential as a delivery carrier of siRNA, tumor antigens, fluorescent proteins, gold nanoparticles, etc., for cancer imaging and/or therapy.^1^, ^2^, ^3^, ^4^, ^5^, ^6^ HBVC is a nanoscale protein particle (named protein nanoparticle, PNP) that is synthesized inside cells with a uniform size, shape, and surface topology. That is, it is synthesized through the self‐assembly of 240 core proteins (subunits) even in *Escherichia coli* and has a stable spherical shape with a diameter of 36 nm.^7^ Recently, other PNPs including viral capsid particles from human papilloma and tobacco mosaic viruses, human ferritin, bacterial proteasome, etc., have attracted much interest as carrier of a variety of anticancer drug agents.^5^, ^8^, ^9^, ^10^, ^11^, ^12^ PNPs have distinct advantages over synthetic nanomaterials such as carbon, metal, and polymer nanoparticles, because they can be easily biosynthesized with a very narrow size distribution; multicopies of heterologous peptides/proteins can be genetically introduced on their surface with preserving native conformation and function; and furthermore they are much more biocompatible. In particular, the genetic insertion of cancer‐targeting peptides into the surface of PNPs enables the modified PNPs to be used as cancer‐targeting carriers of anticancer drugs. Furthermore, recent literatures report that synthetic nanomaterials cause nonspecific in vivo accumulation and accordingly severe nanotoxicity problems.^3^, ^13^, ^14^

Despite the significant advantages stated above, PNPs suffer from an intrinsic drawback, that is, potential immunogenicity problem that needs to be overcome for their successful clinical application as carrier of a variety of drugs except for vaccines.^12^, ^15^, ^16^ Although several researchers have recently reported that the immunogenicity of viral capsids was significantly reduced through modifying a particular sequence,^17^, ^18^ here we tried to resolve the immunogenicity problem through a novel method that is generally applicable to a variety of PNPs, which is based on genetic presentation of ABPs on PNP surface, enabling serum albumins to bind to the modified PNP surface. Serum albumin is the most abundant and nonimmunogenic native protein found in human blood plasma and has a long half‐life inside the bodies.^19^ Moreover, serum albumin accumulates in malignant and inflamed tissues due to their leaky capillary combined with defective lymphatic drainage system. In addition, serum albumin is attracted by tumors because albumin is a major energy and nutrition source for tumor growth.^20^, ^21^ It is therefore suggested that the presentation of ABPs on PNP surface can significantly reduce immunogenicity of PNPs and further enhance efficacy of PNPs as cancer‐targeting drug carrier under in vivo physiological conditions.

In the present study, as an example of PNP we employed HBVC where the immunogenicity results from three major regions of core protein: weakly immunogenic N‐ and C‐terminus and an internal flexible loop that is localized on the most external surface of HBVC and thus forms strongly immunogenic spike‐like conformation (named spike region).^7^ Here, the internal flexible loop of core protein was switched to an ABP with the sequence of DDEWLCGWRPLCIDEILR that was previously identified through phage display studies.^22^, ^23^ The genetic insertion of ABP into the HBV core protein and subsequent expression of the ABP‐modified core proteins in *E. coli* led to the successful synthesis of recombinant HBVC that presents 240 copies of ABP on its external surface (named ABP‐HBVC). We characterized the albumin‐binding and cancer‐targeting properties of ABP‐HBVC and further evaluated its immunogenicity through cytokine and antibody titer assays using live mice that were intravenously or intraperitoneally injected with ABP‐HBVC.

## 2 Result and Discussion

### 2.1 Genetic Insertion of ABPs to the Surface of HBVC and Biosynthesis of ABP‐HBVC

**Figure 1:**
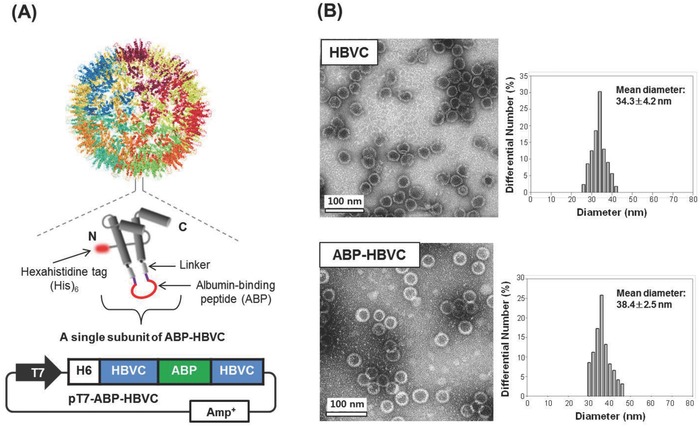
Genetic presentation of ABPs on the surface of HBVC. A) Schematic illustration of ABP‐HBVC and plasmid expression vector used in *E. coli* for the biosynthesis of ABP‐HBVC. B) Results of TEM and DLS analyses of purified HBVC (free of ABPs) and ABP‐HBVC.

An ABP sequence, DDEWLCGWRPLCIDEILR with high binding affinity for both human and mouse serum albumins^22^, ^23^ was inserted into an internal loop region of HBVC core protein (assembly subunit) by replacing the dipeptidyl sequence of Asp79‐Arg80, resulting in the construction of plasmid expression vector (pT7‐ABP‐HBVC) that codes for the synthesis of *NH_2_*‐*Nde*I‐H_6_‐HBVC(1‐78)‐G_4_SG_4_T‐*Xho*I‐ABP‐*Bam*HI‐G_4_SG_4_T‐HBVC(81‐149)‐*Cla*I‐*COOH*, where G_4_SG_4_T is a linker peptide and H_6_ was inserted for Ni^+2^‐affinity purification of ABP‐HBVC (**Figure** **1**A). The plasmid expression vector (pET‐HBVC) for the ABP‐free HBVC synthesis was also prepared as described in Figure S1A (Supporting Information). When expressed using the expression vector, pT7‐ABP‐HBVC in *E. coli*, the ABP‐inserted core proteins were self‐assembled into ABP‐HBVC with a uniform shape and size (38.4 ± 2.5 nm) in the bacterial cytoplasm (Figure 1B, Figure S1B, Supporting Information). The ABP‐free HBVC also has native‐like particle shape and size with a narrow size distribution (34.3 ± 4.2 nm) (Figure 1B).

### 2.2 Characterization of ABP‐HBVC: Albumin‐Binding Activity and Endotoxin Assays

**Figure 2:**
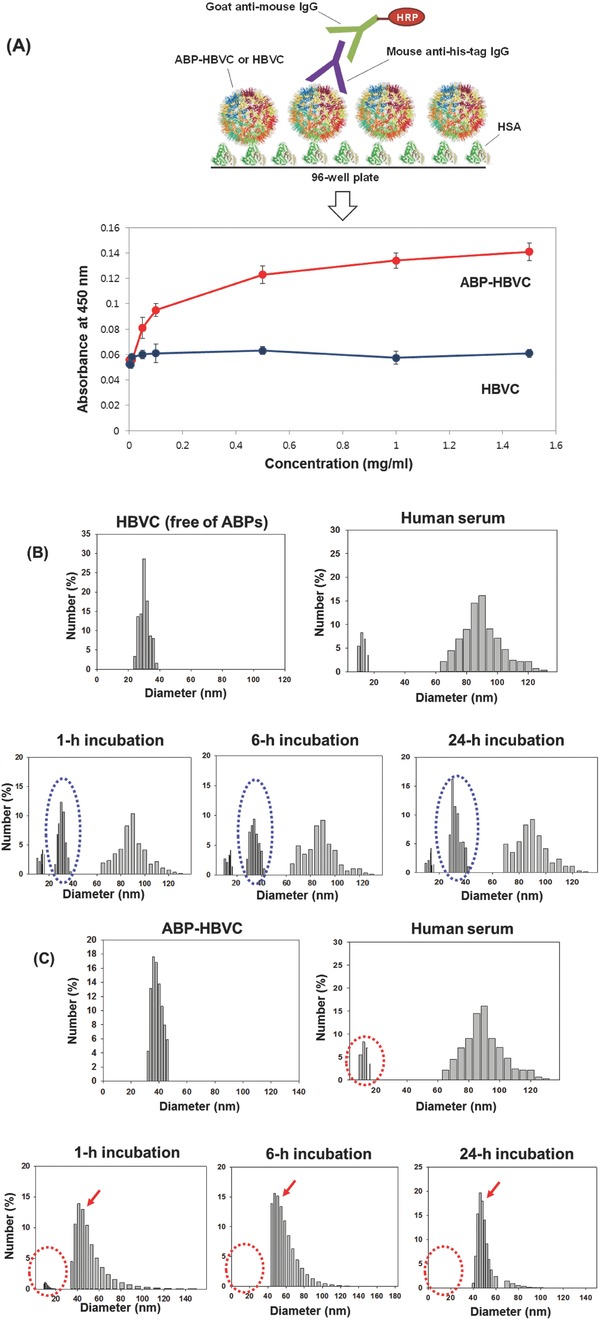
Albumin‐binding activity of ABP‐HBVC. A) Results of ELISA. B) Results of time‐course DLS analysis of the mixture of HBVC (free of ABPs) and human serum. The blue dotted circles represent the DLS peaks for HBVC. C) Results of time‐course DLS analysis of the mixture of ABP‐HBVC and human serum. The red dotted circles and arrows represent the DLS peaks for HSA and agglomerates of HSA‐ABP‐HBVC‐binding complexes, respectively.

We estimated the albumin‐binding activity of ABP‐HBVC through enzyme‐linked immunosorbent assay (ELISA) as presented in **Figure** **2**A, showing that the apparent absorbance signals were observed due to the binding between ABP‐HBVC and human serum albumin (HSA) while the ABP‐free HBVC (negative control) did not generate any HSA‐binding‐associated signals. The albumin‐binding activity of ABP‐HBVC was further monitored through time‐course dynamic light scattering (DLS) analysis after ABP‐HBVC (or ABP‐free HBVC) was added to human serum (Figure 2B,C). The particle size of ABP‐free HBVC (indicated by blue dotted circles in Figure 2B) never changed for 24 h in human serum, while both of the DLS peaks for ABP‐HBVC and HSA (that are present as a dimer having the size of ≈14 nm,^24^, ^25^ indicated by red dotted circles in Figure 2C) disappeared, and the new peaks around 50 nm (indicated by red arrows in Figure 2C) newly appeared immediately after ABP‐HBVC was added to human serum, showing clearly the binding between ABP‐HBVC and serum albumins. We also measured the amount of *E. coli*‐derived endotoxin (LPS) involved in the purified ABP‐HBVC and ABP‐free HBVC. As presented in Figure S3 (Supporting Information), the measured level of endotoxin was less than 10 EU mL^−1^, which is such a negligible level that cannot cause a meaningful immune response in vivo according to the previous reports.^26^, ^27^, ^28^, ^29^

### 2.3 Tumor Targeting and In Vivo Distribution of ABP‐HBVC in Tumor‐Bearing Mice

**Figure 3:**
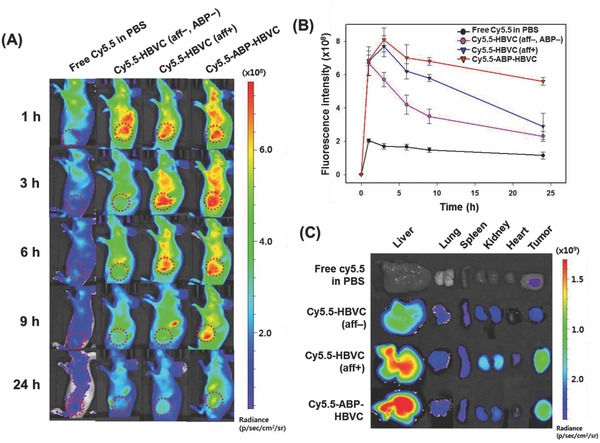
Tumor targeting and biodistribution of ABP‐HBVC in live mice. A) NIR fluorescence images of live mice that were intravenously injected with Cy5.5‐labeled recombinant HBVC particles (HBVC (aff−, ABP−), HBVC (aff+), and ABP‐HBVC). B) Time‐course NIR fluorescence intensity from the tumor in live mice of (A). C) Ex vivo NIR fluorescence images of five major organs and tumor that were excised from live mice of (A) at 48 h after the IV injection.

The tumor‐targeting performance and in vivo distribution of ABP‐HBVC in live mice were monitored at predetermined time points for 24 h after the intravenous administration of Cy5.5‐labeled ABP‐HBVC into U87MG (glioblastoma tumor)‐bearing mice. The term, “tumor‐targeting performance” is defined here as the capability of performing both of the following events: efficient delivery to and stable retainment in tumor. For this study, we synthesized another type of recombinant HBVC particle (HBVC (aff+)) by substituting the ABP sequence of ABP‐HBVC with the tandem sequence of affibody peptide that has strong and specific affinity for human epidermal growth factor receptor I (EGFR) (Figure S2, Supporting Information). EGFR is overexpressed on the cell surface of a wide range of tumors including U87MG.^5^, ^6^ From **Figure** **3**A,B, compared to Cy5.5‐labeled HBVC (aff−, ABP−) (free of both affibody and ABP), a significantly larger amount of Cy5.5‐labeled ABP‐HBVC and HBVC (aff+) was delivered to the tumor (U87MG), and it is further notable that ABP‐HBVC remained in the tumor for a longer period. This suggests that the aforementioned role of serum albumin as a major energy and nutrition source for tumor growth led to the longer retention of ABP‐HBVC in the tumor and accordingly demonstrates higher tumor‐targeting performance of ABP‐HBVC, which corresponds to the previous report of Dennis et al.^22^ that ABP has longer half‐life than other peptides due potentially to the binding of ABP to albumin. The ex vivo fluorescence images of five major organs and tumor show the appreciable accumulation of both ABP‐HBVC and HBVC (aff+) in liver (Figure 3C), which is probably because EGFR is also highly expressed in liver cell surface, and liver is a major albumin‐producing organ. Figure S4 (Supporting Information) shows that compared to ABP‐free HBVC (aff−, ABP−), the much higher amount of ABP‐HBVC accumulated in tumor. In addition, the noticeable amount of HBVC (aff+) was detected in kidney (Figure 3C), suggesting that HBVC (aff+) is more quickly removed from the body through renal excretion than ABP‐HBVC.

### 2.4 Evaluation of Immunogenicity of ABP‐HBVC Using Live Mice through Cytokine and Immunoglobulin (Ig) Titer Assays

**Figure 4:**
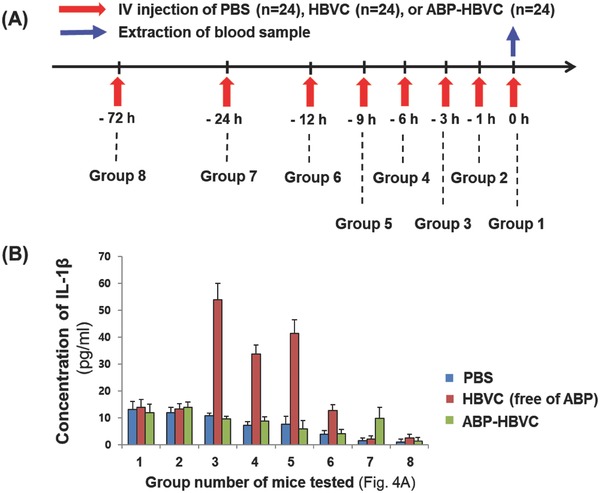
Concentration of serum IL‐1β in live mice injected with ABP‐HBVC and HBVC (free of ABPs). A) Time schedule of IV injection of PBS (negative control), HBVC (50 µg), and ABP‐HBVC (50 µg) to C57BL/6 mice (*n* = 72). B) Results of time‐course ELISA to measure serum IL‐1β in live mice of (A).

In order to evaluate the immunogenicity of ABP‐HBVC, we first measured the serum concentration of interleukin 1β (IL‐1β) from C57BL/6 mice^30^, ^31^ (*n* = 72) that were intravenously (IV) injected with phosphate buffered saline (PBS) (negative control) (*n* = 24), HBVC (free of ABP) (*n* = 24), and ABP‐HBVC (*n* = 24). IL‐1β that is produced from activated macrophages and helper T cells is an important cytokine indicator of inflammatory response.^30^, ^31^, ^32^, ^33^ The serum samples were collected from the live mice above at predetermined points for 72 h after the IV injection of PBS, HBVC (50 µg), and ABP‐HBVC (50 µg), as described in **Figure** **4**A. Before the IV injection of HBVC and ABP‐HBVC, we confirmed that endotoxin (LPS) level in each sample was negligible, as presented in Figure S3 (Supporting Information). From the results of ELISA (Figure 4B), it is obvious that the serum IL‐1β concentration (far lower than 20 pg mL^−1^) in the mice injected with ABP‐HBVC was nearly identical to that of negative control for the entire period of study (72 h), while the concentration of IL‐1β in the HBVC‐injected mice increased to almost four times higher level at 3 h after IV injection and then gradually decreased and reached the level of negative control after 24 h. Compared to the IL‐1β concentrations (500–3000 pg mL^−1^) in the mice injected with various immunogenic viral proteins,^33^, ^34^, ^35^, ^36^, ^37^ the results of Figure 4B indicate that the ABPs presented on HBVC significantly lowers in vivo inflammatory response caused by HBVC due to ABP‐mediated binding of albumins to HBVC.

**Figure 5:**
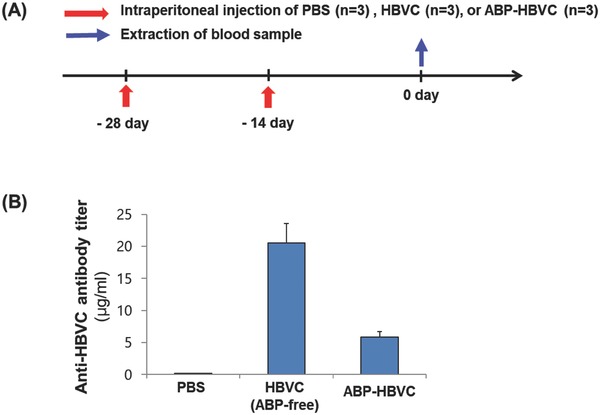
Anti‐HBVC antibody titer in live mice injected with ABP‐HBVC and HBVC (free of ABPs). A) Time schedule of intraperitoneal injection of PBS (negative control), HBVC (50 µg), and ABP‐HBVC (50 µg) to C57BL/6 mice (*n* = 9). B) Results of ELISA to measure anti‐HBVC antibody (immunoglobulins except for IgM) titer in live mice of (A).

We also estimated Ig titers (except for IgM) in the sera of live mice (C57BL/6) (*n* = 9) that were intraperitoneally injected twice with PBS (*n* = 3), HBVC (free of ABP) (50 µg) (*n* = 3), and ABP‐HBVC (50 µg) (*n* = 3) (**Figure** **5**A). We also confirmed the negligible level of endotoxin (LPS) in the injected samples. As seen from Figure 5B, the Ig titer (around 5 µg mL^−1^, measured through ELISA of the collected sera) in the live mice injected with ABP‐HBVC was much lower than that in the HBVC‐injected mice, which is far lower than the previously reported antibody (IgG) titer values (50–100 µg mL^−1^) in the mice injected with various immunogenic viral proteins.^38^, ^39^, ^40^ This indicates again that the immunogenicity of HBVC was significantly reduced by the ABP presentation on its surface. Therefore, replacing the immunogenic spike region of HBVC with ABP seems highly effective in reducing its immunogenicity.

## 3 Conclusion

PNPs have a great potential as drug delivery carrier because they offer significant advantages over conventional synthetic nanomaterials in the aspect of functional versatility for drug conjugation or loading, in vivo biocompatibility, and consistency of material quality. PNPs are found in a broad range of living organisms from microorganism such as virus and bacteria to human and have highly diverse structural characteristics in terms of size, shape, assembly pattern, and surface topology.^41^, ^42^ Each type of PNP is biosynthesized inside cells through its own self‐assembly pattern, which always enables nanoparticle synthesis in a highly reproducible manner. Furthermore, PNPs are spontaneously disassembled under in vivo physiological conditions and hence do not cause long‐term accumulation‐associated toxicity problems.^5^, ^6^ In general, the PNP surface is easily engineered through a simple genetic modification of subunit protein, which enables efficient conjugation or loading of a variety of cancer diagnostic and therapeutic agents such as siRNA, metal nanoparticles, fluorescent proteins, small synthetic molecules, etc., to the genetically modified PNP surface.^3^, ^4^, ^5^, ^6^, ^10^ Recently, superparamagnetic gold nanoparticle cluster (SPAuNC) was synthesized on the modified surface of HBVC where the peptide ligands for targeting cancer cells and chemisorbing gold ions are genetically presented. SPAuNC was successfully applied to in vivo cancer targeted theragnosis (i.e., both magnetic resonance imaging and magnetic hyperthermia therapy of cancer) followed by undergoing rapid renal clearance,^6^ where the half‐life of HBVC carrier was ≈12 h. This long half‐life is probably due to a much longer circulating half‐life of HBVC compared to other viral particles such as cowpea mosaic virus, cowpea chlorotic mottle virus, and tobacco mosaic virus (3–12 min).^43^, ^44^, ^45^, ^46^ Despite these superior advantages, PNPs suffer from an intrinsic drawback that hampers their clinical application, that is, potential immunogenicity. Here, we report a novel method for resolving the immunogenicity problem of PNPs, which is based on the genetic presentation of ABPs on the surface of PNP (e.g., HBVC used in this study). That is, we replaced the immunogenic regions of HBVC with ABPs without interfering self‐assembly activity of HBVC. The ABPs on the HBVC surface show apparent activity of gathering human serum albumins around HBVC, which significantly reduced in vivo inflammatory response and immunoglobulin titer compared to ABP‐free HBVC. Furthermore, the ABP‐HBVC retained in tumor for much longer period of time, compared to the ABP‐free HBVC, which reportedly seems to be due to the long half‐life of ABP that binds to serum albumin that is a major energy and nutrition source for tumor growth. This cancer‐targeting capability of ABP can be applied to other PNP‐based drug carriers like SPAuNC,^6^ which would be achieved through replacing the cancer‐targeting affibodies with ABP. This novel approach was applied to HBVC as a proof of concept here but may be used as a general platform for resolving immunogenicity and cancer‐targeting problems of PNPs capable of delivering a broad range of drug agents, which offers considerable advantages over traditional synthetic nanomaterials in developing new drug delivery carriers with high safety and efficacy.

## 4 Experimental Section

*Biosynthesis and Characterization of Recombinant Capsid Particles: HBVC, HBVC (aff+), and ABP‐HBVC*: Through polymerase chain reaction (PCR) amplification using the appropriate primers, the three gene clones were prepared, encoding *NH_2_*‐*Nde*I‐H_6_‐HBVC‐*Hin*dIII‐*COOH*, *NH_2_*‐H_6_‐biotinylated peptide‐G_3_SG_3_TG_3_SG_3_Y_6_‐HBVC(1‐78)‐G_4_SG_4_T‐(affibody peptide)_2_‐G_4_S G_4_T‐HBVC(81‐149)‐*COOH*, and *NH_2_*‐*Nde*I‐H_6_‐HBVC(1‐78)‐G_4_SG_4_T‐*Xho*I‐ABP(DDEWLCGWRPLCIDEILR)‐*Bam*HI‐G_4_SG_4_T‐HBVC(81‐149)‐*Cla*I‐*COOH*, and sequentially ligated into pT7‐7 or pET28a plasmid to construct the following expression vectors: pET‐HBVC, pT7‐aff‐HBVC, and pT7‐ABP‐HBVC, respectively (Figure 1, Figures S1 and S2, Supporting Information). The affibody peptide above has specific and strong affinity for human epidermal growth factor receptor I (EGFR). After complete sequencing, *E. coli* BL21 (DE3) was transformed with each of pET‐HBVC, pT7‐aff‐HBVC, and pT7‐ABP‐HBVC, and ampicillin‐ or kanamycin‐resistant transformants were selected and used to synthesize the HBVC, HBVC (aff+), and ABP‐HBVC. After cultivated at 20 °C for 16 h following IPTG (isopropyl β‐d‐1‐thiogalactopyranoside) induction (1 × 10^−3^ m), the recombinant *E. coli* cells were harvested, followed by purification of recombinant capsid particles as described in previous reports.^3^, ^4^, ^5^, ^6^, ^10^ Sodium dodecyl sulfate‐polyacrylamide gel electrophoresis (SDS‐PAGE) of the each purified capsid particle revealed a single protein band corresponding to the cognate subunit protein.

Size distribution of the prepared recombinant capsid particles was measured in triplicate using a Zetasizer Nano ZS (Malvern Instruments, Ltd., Worcestershire, UK) equipped with a 633 nm wavelength laser. The morphological shape of recombinant capsid particles was analyzed using transmission electron microscopy (TEM) (CM‐200 electron microscope, Philips, CA), operating at an 80 kV acceleration voltage. For the preparation of TEM samples, one drop of each capsid particle suspension (1 mg mL^−1^) was placed onto a 200 mesh copper grid precoated with carbon. After 2 min of deposition, distilled water was removed by air‐drying. Negative staining was applied using a droplet of a 2% (w/v) aqueous uranyl acetate solution.

*Near‐Infrared (NIR) Fluorescence Labeling of Recombinant HBVC Particles*: For the estimation of in vivo biodistribution, the three different recombinant capsid particles (HBVC, HBVC (aff+), and ABP‐HBVC) were labeled by NIR dye (Cy5.5) as described below: 2 mmol of Cy5.5 N‐hydroxysuccinimide (NHS) ester (Cy5.5‐NHS, GE Healthcare, Piscataway, NJ) with excitation and emission wavelengths of 675 and 693 nm, respectively, was incubated with each purified recombinant capsid in sodium bicarbonate (0.1 m, pH 8.5) at room temperature for 12 h. The Cy5.5‐labeled recombinant capsid particles were loaded onto a sucrose step gradient (60, 50, 40, 30, and 20 w/v%) and centrifuged at 35 000 rpm for 16 h at 4 °C to separate the unbound/free Cy5.5 from the Cy5.5‐labeled capsids. Subsequently, a fraction of sucrose solution (40–50 w/v% sucrose) containing the Cy5.5‐labeled recombinant capsid particles was collected, followed by buffer exchange to PBS (2.7 × 10^−3^ m KCl, 137 × 10^−3^ m NaCl, 2 × 10^−3^ m KH_2_PO_4_, 10 × 10^−3^ m Na_2_HPO_4_, pH 7.4) through ultrafiltration (Amicon Ultra 10K, Millipore, Billerica, MA).

*Assays of Affinity for HSA and Endotoxin of ABP‐HBVC*: Human serum albumin (Sigma) was immobilized onto NUNC MAXISORP 96‐well plates at 2 µg mL^−1^ overnight at 4 °C. The plates were blocked with Superblock Blocking buffer (Thermo Fisher Scientific) for 1 h at 25 °C. The recombinant capsid particles (ABP‐free HBVC and ABP‐HBVC) at various concentrations (0–1.5 mg mL^−1^, 100 mL) were added to each well with the immobilized HSA, followed by incubation for 1 h at 25 °C. From Figure 2C, it seems clear that the 1 h incubation is sufficient for the complete binding of ABP‐HBVC to HSA. After the unbound recombinant capsid particles were removed by washing steps using 0.05% PBS/Tween‐20, the binding between HSA and recombinant capsid particles were examined through ELISA using mouse anti‐his‐tag (H_6_) IgG and goat anti‐mouse IgG conjugated with horseradish peroxidase (HRP) (Santa Cruz Biotechnology Inc., CA) as primary and secondary antibodies, respectively. The absorbance signals were read at 450 nm. All the ELISA assays were performed in triplicate.

The HSA‐binding activity of ABP‐HBVC was further confirmed through the incubation of ABP‐HBVC in human sera (50 v/v% in PBS (137 × 10^−3^ m NaCl, 2.7 × 10^−3^ m KCl, 10 × 10^−3^ m Na_2_HPO_4_, 2 × 10^−3^ m KH_2_PO_4_, pH 7.4) at 37 °C, followed by DLS analysis of the incubation mixtures at predetermined time points for 24 h. Finally, *E. coli* endotoxin (lipopolysaccharide/LPS) level in the purified recombinant capsid particles was measured by Limulus Amebocyte Lysate (LAL) assay kit (GenScript, NJ, USA) according to the supplier's instruction.

*Evaluation of In Vivo Tumor Targeting Performance of HBVC, HBVC (aff+), and ABP‐HBVC*: All experiments with live mice to evaluate tumor‐targeting performance were performed at Korea Institute of Science and Technology (KIST) in compliance with the relevant laws and institutional guidelines of KIST. U87MG (2 × 10^6^ cells/mouse) suspended in PBS (100 µL) was subcutaneously injected into the flank of athymic nude mice (20 g, Orient, Seoul, Korea). When the size of tumors reached ≈5 mm in diameter, Cy5.5‐labeled recombinant capsid particles (HBVC, HBVC (aff+), or ABP‐HBVC) (50 µg each in 100 µL of PBS) and free Cy5.5 (in 100 µL of PBS) were intravenously injected via a tail vein into the mice (*n* = 12). Prior to the injection, the fluorescence intensity of all samples was adjusted to an equal value. Fluorescence images were noninvasively acquired using an animal imaging system (eXplore Optix System, Advanced Research Technologies Inc., Montreal, Canada) at multiple time points. Laser power and exposure time were optimized at 45 µW and 0.3 s per point, respectively. The NIR fluorescence from tumor was calculated through the region of interest (ROI) analysis using Analysis Workstation software (ART Advanced Research Technologies Inc.). The fluorescence intensity was recorded as total photons per centimeter squared per steradian (p s^−1^ cm^−2^ sr^−1^) per tumor. At 48 h postinjection, the tumors and five major organs (liver, lung, spleen, kidney, and heart) were excised and imaged using the same animal imaging system (eXplore Optix System, Advanced Research Technologies Inc., Montreal, Canada) with Cy5.5 emission filter sets (680–720 nm; Omega Optical, Brattleboro, VT).

*Measurement of IL‐1β Concentration in the Sera of Live Mice Injected with PBS, HBVC, and ABP‐HBVC*: All experiments with live mice to measure serum IL‐1β concentration were performed at Graduate School of Medicine, Korea University (KU) in compliance with Korea University Institutional Animal Care and in accordance with recommendations for proper use and care of laboratory animals (KOREA‐2017‐0137). For the experiment, the sera of healthy mice (C57BL/6) that were intravenously injected with PBS (100 µL, negative control) (*n* = 24), HBVC (50 µg in 100 µL of PBS) (*n* = 24), and ABP‐HBVC (50 µg in 100 µL of PBS) (*n* = 24) were collected at multiple time points for 72 h (i.e., 0, 1, 3, 6, 9, 12, 24, and 72 h). Any of HBVC and ABP‐HBVC were not fluorescence‐labeled. The ELISA kit to measure mouse IL‐1β concentration (mouse IL‐1β ELISA Ready‐SET‐Go!, Cat. No. 88‐7013‐22, eBioscience, Wien, Austria) was used with the following procedure. Each well of Nunc Maxisorp ELISA plate (96‐well plate) was coated with capture antibody (anti‐mouse IL‐1β IgG) in 1× coating buffer (100 µL) overnight at 4 °C. After washed with 1× PBS and Tween‐20 (0.05%), the 96‐well plate was blocked with 1× ELISA/ELISPOT Diluent (200 µL). The collected mouse sera were then added to the wells (100 µL per well), and the plate was sealed and incubated overnight at 4 °C. After adding the HRP‐conjugated reporter antibody and 1× TMB (3,3′,5,5′‐tetramethylbenzidine) solution to each well, followed by adding stop solution (1 m H_3_PO_4_), assay signals (absorbance at 450 nm) were measured using microplate reader (Infinite M200 PRO, TECAN, Austria) and converted the absorbance to pg/mL based on the predetermined correlation. The error bars are standard deviations between groups.

*Measurement of Immunoglobulin Titer in the Sera of Live Mice Injected with PBS, HBVC, and ABP‐HBVC*: All experiments with live mice to measure serum IgG titer were performed at Graduate School of Medicine, Korea University in compliance with Korea University Institutional Animal Care and in accordance with recommendations for proper use and care of laboratory animals (KOREA‐2017‐0137). Female C57BL/6 mice (6–8 weeks of age) were immunized twice with two‐week interval through the intraperitoneal injection of PBS (100 µL, negative control) (*n* = 3), HBVC (50 µg in 100 µL of PBS) (*n* = 3), and ABP‐HBVC (50 µg in 100 µL of PBS) (*n* = 3). (Any of HBVC and ABP‐HBVC were not fluorescence‐labeled.) At two week after the second immunization, each mouse blood was collected through cardiac puncture, followed by the centrifugation of blood at 13 000 rpm for 10 min to prepare the sera.

For measuring the anti‐HBVC immunoglobulin titer in the mouse sera, each well of Nunc Maxisorp ELISA plate (Cat. No. 44‐2404‐21, Thermo Fisher Scientific, MA, USA) was coated with HBVC (2 µg mL^−1^) through overnight incubation at 4 °C. After washed with 1× PBS and Tween‐20 (0.05%), each well was blocked with Superblocking buffer (200 µL) (cat. No. 37515, Thermo Fisher Scientific, MA, USA) for 2 h. The collected serum (100 µL) was then added to each well, and the plate was sealed and incubated overnight at 4 °C. After adding HRP‐conjugated goat anti‐mouse IgG (Cat. No. AP127P, Sigma Aldrich, St. Louis, MO) that can recognize all mouse immunoglobulins except for IgM and 1× TMB solution to each well, followed by adding stop solution (1 m H_3_PO_4_), anti‐HBVC immunoglobulin titers (i.e., absorbance at 450 nm) were measured using microplate reader (Infinite M200 PRO, TECAN, Austria) and converted the absorbance to µg/mL based on the predetermined correlation. All data were presented as means ± standard deviation.

## Conflict of Interest

The authors declare no conflict of interest.

## Supporting information

Supplementary
