# Supplementary material for: Nonimmunogenetic Viral Capsid Carrier with Cancer Targeting Activity
Source: Adv Sci (Weinh). 2018 Jun 27;5(8):1800494. doi: 10.1002/advs.201800494 (PMC6097151; doi:10.1002/advs.201800494)
Supplement: Supplementary file 1 — Supplementary [file ADVS-5-1800494-s001.pdf]

Copyright WILEY-VCH Verlag GmbH & Co. KGaA, 69469 Weinheim, Germany, 2018.

ADVANCED SCIENCE Open Access

Supporting Information

for *Adv. Sci.,* DOI: 10.1002/advs.201800494

Nonimmunogenetic Viral Capsid Carrier with Cancer Targeting Activity

*Bo-Ram Lee, Eunji Jo, Hong Yeol Yoon, Chul Joo Yoon, Hyo-Jung Lee, Koo Chul Kwon, Tae Woo Kim, and Jeewon Lee**

Copyright WILEY-VCH Verlag GmbH & Co. KGaA, 69469 Weinheim, Germany, 2016.

Supporting Information

Non-immunogentic viral capsid carrier with cancer targeting activity

Bo-Ram Lee[1], Eunji Jo[1], Hong Yeol Yoon[2], Chul Joo Yoon[1], Hyo-Jung Lee[3], Koo Chul Kwon[1], Tae Woo Kim[3], and Jeewon Lee[1]*

[*]Prof. J. Lee(Corresponding author), B.-R. Lee, E. Jo, C. J. Yoon, Dr. K. C. Kwon
Department of Chemical and Biological Engineering, College of Engineering,
Korea University, Anam-Ro 145, Seoul 136-713 (Republic of Korea)
E-mail:leejw@korea.ac.kr

Dr. Y. H. Yoon
Center for Theragnosis, Biomedical Research Institute, Korea Institute of Science and Technology, 39-1 Hawolgok-dong, Seongbuk-gu, Seoul 136-791 (Republic of Korea)

Prof. T. W. Kim, H.-J. Lee
Division of Infection and Immunology, Graduate School of Medicine,
Korea University, Anam-Ro 145, Seoul 136-713 (Republic of Korea)

Revised manuscript (advs. 201800494) submitted to

Advanced Science

Supplementary Figures

Figure S1

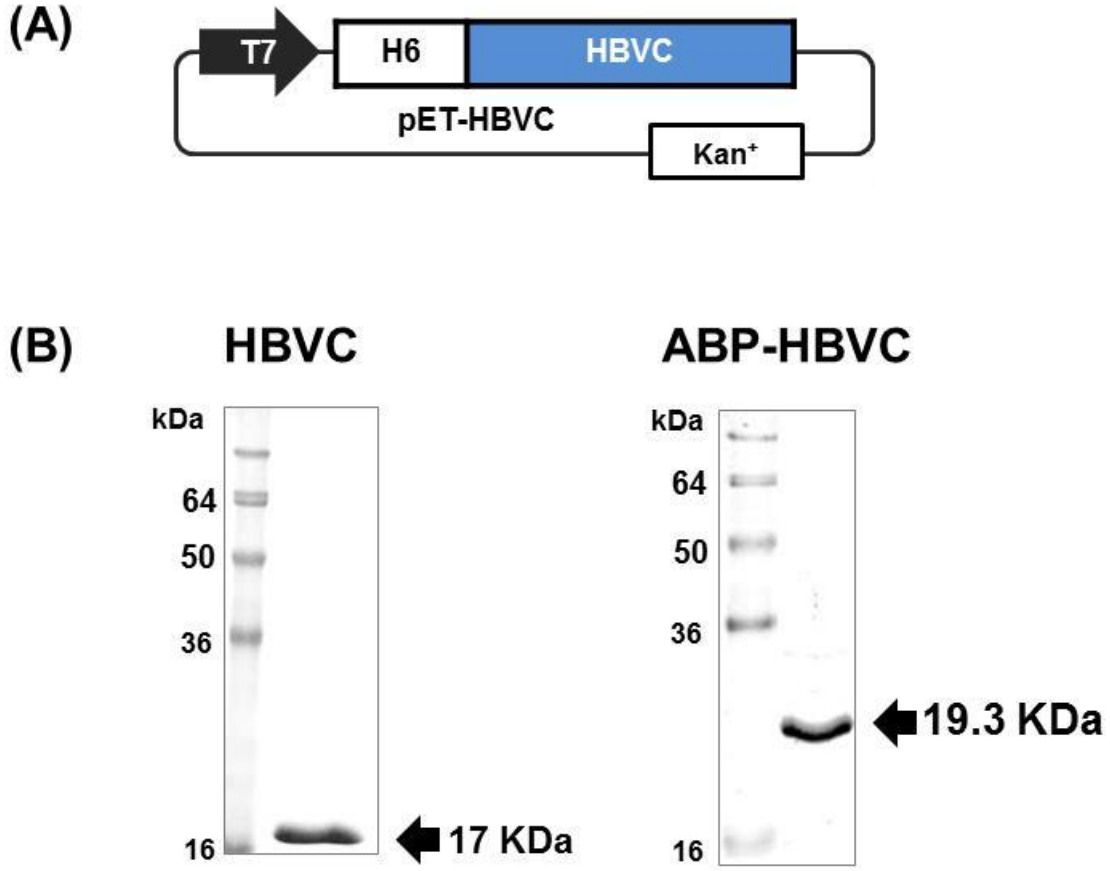

Figure S1. (A) Plasmid expression vector used in *E. coli* to synthesize HBVC (free of ABPs). **(B)** Result of SDS-PAGE of the purified HBVC and ABP-HBVC.

Figure S2

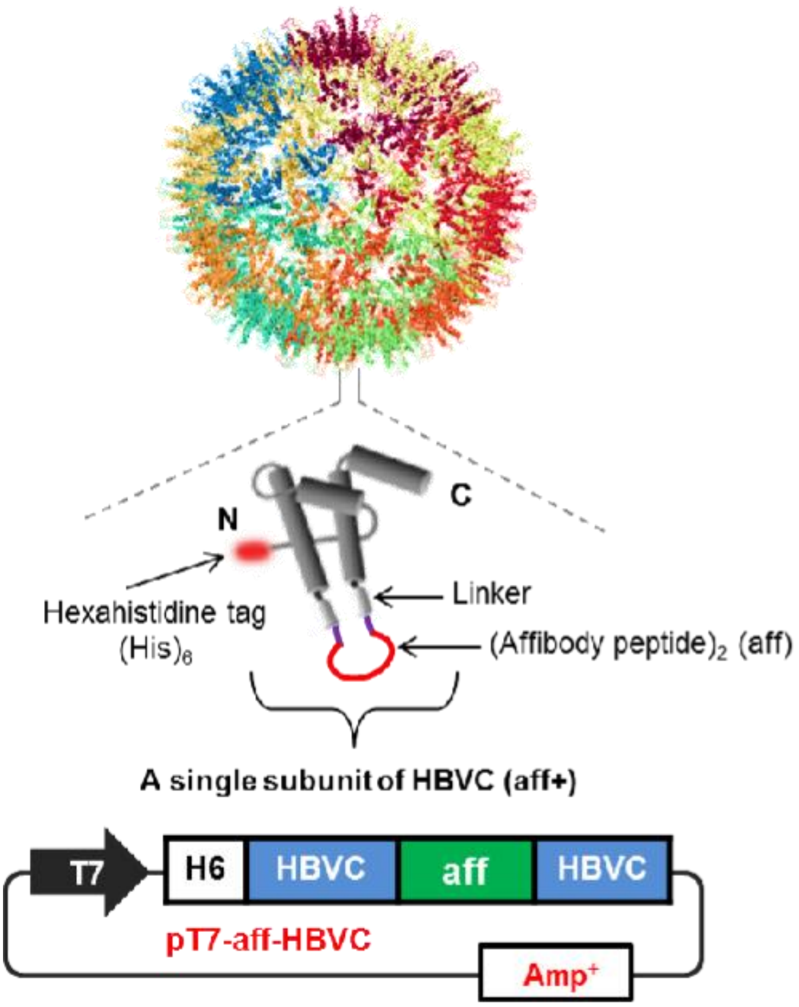

Figure S2. Schematic illustration of HBVC (aff+) and plasmid expression vector used in *E. coli* to synthesize HBVC (aff+)**.**

Figure S3

(A)

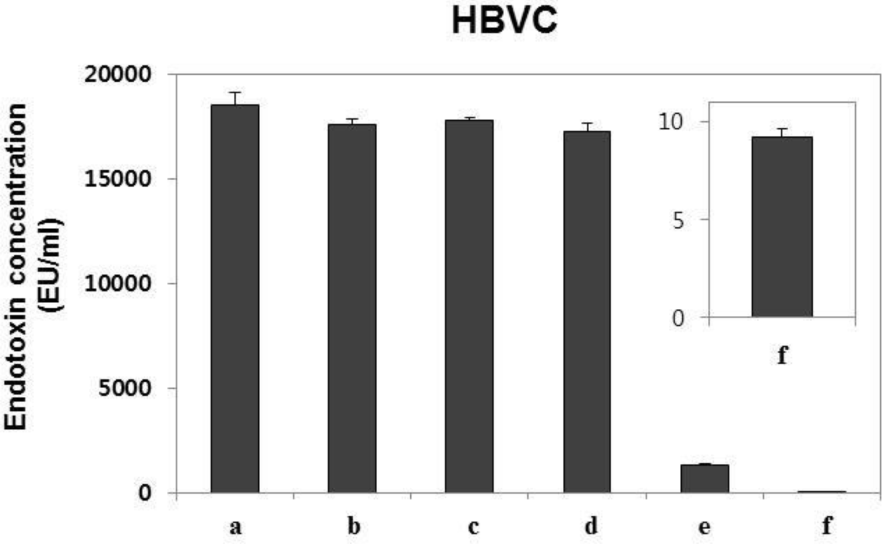

(B)

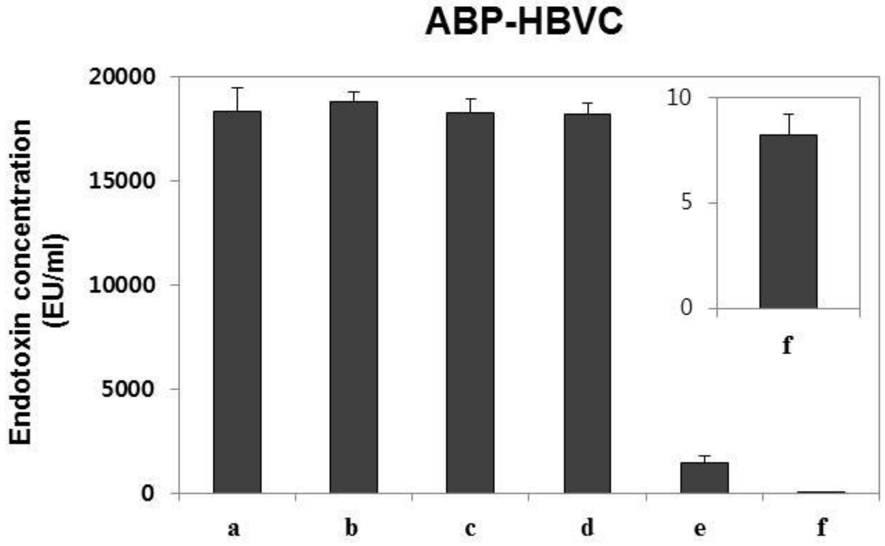

Figure S3. Results of endotoxin assays of the purified HBVC (free of ABPs) (**A**) and ABP-HBVC (**B**), using crude lysate of recombinant *E. coli* cells **(a)**, supernatant of centrifuged cell lysate of a (**b**), flow-through from Ni^{+2}-affinity column loaded with supernatant of b (**c**), flow-through after washing Ni^{+2}-affinity column of c (**d**), eluted solution from Ni^{+2}-affinity column of d (**e**), and purified ABP-HBVC or ABP-free HBVC after sucrose gradient fractionation of eluted solution of e (**f**).

Figure S4

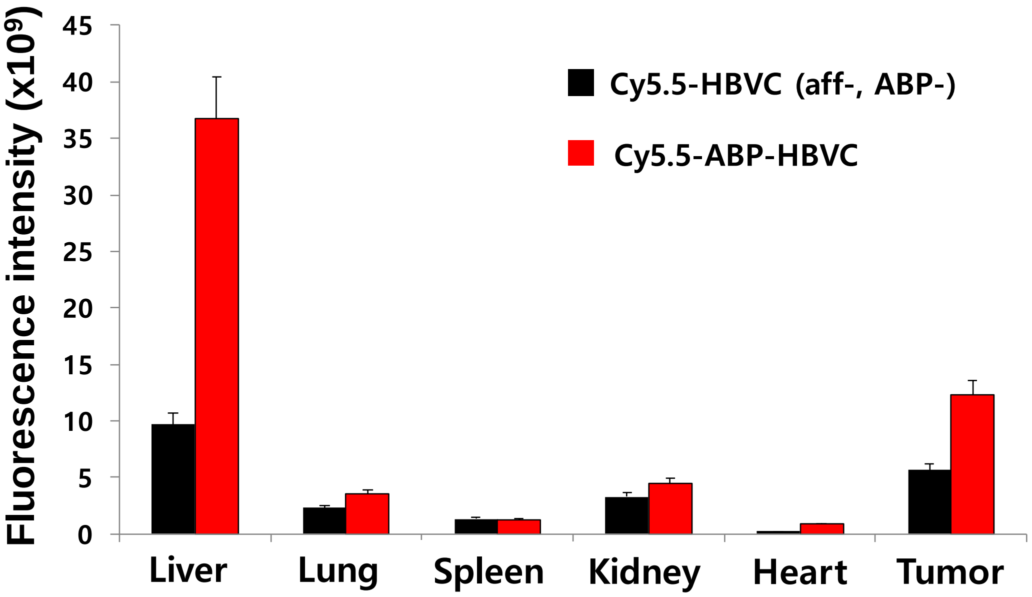

Figure S4. *Ex vivo* NIR fluorescence intensities (spatial scale bar) of five major organs and tumor that were excised from live mice that were intravenously injected with ABP-free HBVC and ABP-HBVC.
